# Rosiglitazone has a neutral effect on the risk of dementia in type 2 diabetes patients

**DOI:** 10.18632/aging.101944

**Published:** 2019-05-14

**Authors:** Chin-Hsiao Tseng

**Affiliations:** 1 Department of Internal Medicine, National Taiwan University College of Medicine, Taipei, Taiwan; 2 Division of Endocrinology and Metabolism, Department of Internal Medicine, National Taiwan University Hospital, Taipei, Taiwan; 3 Division of Environmental Health and Occupational Medicine of the National Health Research Institutes, Zhunan, Taiwan

**Keywords:** dementia, diabetes mellitus, rosiglitazone, Taiwan

## Abstract

This study investigated whether rosiglitazone might increase or reduce dementia risk. Taiwan’s National Health Insurance database was used to enroll a cohort of 1:1 matched-pairs of ever and never users of rosiglitazone based on propensity score from patients with new-onset type 2 diabetes during 1999-2006. The patients were alive on January 1, 2007 and were followed up for dementia until December 31, 2011. A total of 5,048 pairs of never users and ever users were identified. The incident case numbers were 127 and 121, respectively. The adjusted hazard ratio for ever versus never users was 0.895 (95% confidence interval: 0.696-1.151). The adjusted hazard ratios for the first (<12.1 months), second (12.1-25.1 months) and third (>25.1 months) tertiles of cumulative duration of rosiglitazone therapy were 0.756 (0.509-1.123), 0.964 (0.685-1.357) and 0.949 (0.671-1.341), respectively. When cumulative duration was treated as a continuous variable, the adjusted hazard ratio was 1.000 (0.992-1.008). Subgroup analyses conducted in ever users and never users of metformin and in patients diagnosed with diabetes during three different periods of time, i.e., 1999-2000, 2001-2003 and 2004-2006, all supported a neutral effect of rosiglitazone. In conclusion, rosiglitazone does not increase or redcue the risk of dementia.

## Introduction

Dementia is a clinical presentation characterized by progressive deterioration of cognitive functions such as memory, thinking and reasoning and behavioral abilities for daily life and self-care. Dementia can result from a vascular etiology or a neurodegenerative disease known as Alzheimer’s disease. Insulin resistance in the brain can be observed in patients with Alzheimer’s disease [1–3]. Some experts coined the term “type 3 diabetes” in 2005 [1] to reflect the close link between diabetes mellitus and Alzheimer’s disease, because they share potential common pathophysiological changes of impaired insulin expression and insulin resistance [1–3]. The increased risk of dementia in diabetes patients may be explained by some pathophysiological changes related to diabetes mellitus that lead to atherosclerosis and neurodegeneration, including insulin resistance, increased inflammation and oxidative stress, deposition of advanced glycation end-products and lipid dysregulation [4].

Increased deposition of amyloid beta (Aβ) and hyperphosphorylation of Tau protein are important pathological changes in the brain of patients with Alzheimer’s disease [2]. Aβ is formed by cleaving the amyloid precursor protein by secretases [5]. Peroxisome proliferator-activator receptor gamma (PPARγ) is expressed in the brain [6] and it downregulates the expression of secretase and reduces Aβ deposition [7]. Knockdown of the PPARγ gene may also affect the expression of several other genes associated with Alzheimer’s disease [8]. Therefore, drugs that improve insulin resistance or activate PPARγ in the brain can theoretically be beneficial in preventing Alzheimer’s disease or dementia [2,9]. In our previous studies, two antidiabetic drugs, specifically metformin and pioglitazone (a PPARγ agonist), that improve insulin resistance and cross the blood-brain-barrier [10,11], reduced the risk of dementia in a dose-response pattern in patients with type 2 diabetes mellitus [12,13].

Rosiglitazone is another PPARγ agonist that had been commonly used as an oral antidiabetic drug before 2007 but is now rarely used because of a potential risk of cardiovascular disease [14]. Whether rosiglitazone might increase or reduce the risk of dementia has rarely been studied. Some *in vitro*, *in vivo* and animal studies suggested a neuroprotective effect of rosiglitazone [15–23]. An early pilot clinical trial conducted in 30 subjects (20 assigned to rosiglitazone and 10 assigned to placebo) with mild Alzheimer’s disease or amnestic mild cognitive impairment suggested that rosiglitazone use for 6 months might have a potential for the treatment of cognitive decline [24]. However, this beneficial effect of rosiglitazone could not be confirmed by later clinical trials [25–27].

By using a cohort of 1:1 propensity score matched-pairs of rosiglitazone ever users and never users derived from the reimbursement database of Taiwan’s National Health Insurance (NHI), the present study investigated whether rosiglitazone could increase or reduce dementia risk in patients with type 2 diabetes. In data analyses, ever users of pioglitazone were excluded and the potential confounding effect of metformin was addressed by subgroup analyses in ever users and never users of metformin.

## RESULTS

### Baseline characteristics

The characteristics of a selected cohort of 1:1 propensity score matched-pairs of never and ever users of rosiglitazone are shown in Table 1. The two groups were well matched and none of the calculated values of the standardized difference between ever and never users of rosiglitazone was found to be >10%.

**Table 1:** Characteristics in never and ever users of rosiglitazone.

Variable	Never users			Ever users		
	(n=5048)			(n=5048)		Standardized difference
	n	%		n	%	
**Demographic data**						
Age (years)	61.10	10.17		61.21	9.77	0.88
Sex (men)	2740	54.28		2759	54.66	0.91
Diabetes duration (years)	5.62	2.46		5.63	2.07	0.46
Occupation						
I	2259	44.75		2246	44.49	
II	1091	21.61		1081	21.41	-0.33
III	855	16.94		828	16.40	-1.55
IV	843	16.70		893	17.69	2.48
Living region						
Taipei	1966	38.95		1964	38.91	
Northern	467	9.25		461	9.13	-0.36
Central	1457	28.86		1424	28.21	-1.50
Southern	542	10.74		545	10.80	0.38
Kao-Ping and Eastern	616	12.20		654	12.96	2.35
**Major comorbidities**						
Hypertension	3717	73.63		3712	73.53	-0.43
Dyslipidemia	3751	74.31		3765	74.58	0.37
Obesity	244	4.83		232	4.60	-1.19
**Diabetes-related complications**						
Nephropathy	949	18.80		943	18.68	-0.31
Eye disease	1339	26.53		1365	27.04	1.14
Stroke	1067	21.14		1016	20.13	-2.75
Ischemic heart disease	1881	37.26		1846	36.57	-1.60
Peripheral arterial disease	962	19.06		971	19.24	0.34
**Major risk factors of dementia**						
Head injury	71	1.41		48	0.95	-4.74
Parkinson's disease	38	0.75		42	0.83	0.78
Hypoglycemia	70	1.39		81	1.60	1.65
Atrial fibrillation	99	1.96		107	2.12	1.07
**Potential risk factors of cancer**						
Chronic obstructive pulmonary disease	1850	36.65		1861	36.87	0.26
Tobacco abuse	91	1.80		93	1.84	0.09
Alcohol-related diagnoses	235	4.66		216	4.28	-1.75
**Antidiabetic drugs**						
Insulin	203	4.02		203	4.02	0.07
Sulfonylurea	3654	72.39		3720	73.69	3.25
Metformin	3510	69.53		3462	68.58	-2.39
Meglitinide	353	6.99		326	6.46	-2.11
Acarbose	591	11.71		604	11.97	0.71
**Medications commonly used in diabetes patients**						
Angiotensin converting enzyme inhibitor/angiotensin receptor blocker	3309	65.55		3296	65.29	-0.75
Calcium channel blocker	2478	49.09		2474	49.01	-0.40
Statins	3112	61.65		3094	61.29	-0.71
Fibrates	1915	37.94		1893	37.50	-0.92
Aspirin	2646	52.42		2683	53.15	1.22
**Oral anticoagulant**						
Warfarin	106	2.10		107	2.12	0.14

Age and diabetes duration are shown as mean and standard deviation.

### Incidences of dementia and hazard ratios by rosiglitazone exposure

The incidence rates of dementia and the hazard ratios by rosiglitazone exposure are shown in Table 2. After a median follow-up of 4.8 years in either the ever users or never users of rosiglitazone, there were 127 incident cases of dementia in never users and 121 incident cases in ever users. The incidence rates of dementia were 616.79 and 537.54 per 100,000 person-years, respectively. The adjusted hazard ratio for ever versus never users of rosiglitazone was 0.895 (95% confidence interval: 0.696-1.151). Analyses with cumulative duration of rosiglitazone therapy categorized into tertiles or treated as a continuous variable all favored a neutral effect of rosiglitazone.

**Table 2:** Incidence rates of dementia and hazard ratios by rosiglitazone exposure.

Rosiglitazone use	*n*	*N*	Person-years	Incidence rate (per 100,000 person-years)	HR	95% CI	*P* value
Never users	127	5048	20590.32	616.79	1.000		
Ever users	121	5048	22510.11	537.54	0.895	(0.696-1.151)	0.3878
Tertiles of cumulative duration of rosiglitazone therapy (months)							
Never users	127	5048	20590.32	616.79	1.000		
<12.1	31	1608	7023.11	441.40	0.756	(0.509-1.123)	0.1664
12.1-25.1	45	1725	7668.85	586.79	0.964	(0.685-1.357)	0.8339
>25.1	45	1715	7818.15	575.58	0.949	(0.671-1.341)	0.7654
Cumulative duration of rosiglitazone therapy treated as a continuous variable							
For every 1-month increment of rosiglitazone use					1.000	(0.992-1.008)	0.9954

*n*: incident cases of dementia, *N*: cases followed, HR: hazard ratio, CI: confidence interval

### Subgroup analyses with regards to metformin use

Table 3 shows the results analyzed separately in ever users and never users of metformin. All analyses showed a non-significant effect of rosiglitazone in terms of overall hazard ratios, hazard ratios for the tertiles and hazard ratios for the cumulative duration of rosiglitazone therapy being treated as a continuous variable.

**Table 3:** Subgroup analyses with regards to metformin use for incidence rates of dementia and hazard ratios by rosiglitazone exposure.

Metformin use/rosiglitazone use	*n*	*N*	Person-years	Incidence rate (per 100,000 person-years)	HR	95% CI	*P* value
**Metformin ever users**							
Rosiglitazone never users	81	3510	14374.44	563.50	1.000		
Rosiglitazone ever users	77	3462	15387.74	500.40	0.931	(0.677-1.279)	0.6583
Tertiles of cumulative duration of rosiglitazone therapy (months)							
Never users	81	3510	14374.44	563.50	1.000		
<12.1	16	1138	4952.21	323.09	0.606	(0.352-1.044)	0.0709
12.1-25.1	31	1193	5293.35	585.64	1.092	(0.718-1.660)	0.6824
>25.1	30	1131	5142.18	583.41	1.075	(0.700-1.650)	0.7417
Cumulative duration of rosiglitazone therapy treated as a continuous variable							
For every 1-month increment of rosiglitazone use					1.004	(0.994-1.015)	0.4130
							
**Metformin never users**							
Rosiglitazone never users	46	1538	6215.87	740.04	1.000		
Rosiglitazone ever users	44	1586	7122.37	617.77	0.823	(0.535-1.267)	0.3768
Tertiles of cumulative duration of rosiglitazone therapy (months)							
Never users	46	1538	6215.87	740.04	1.000		
<12.1	15	470	2070.90	724.32	0.996	(0.547-1.815)	0.9906
12.1-25.1	14	532	2375.50	589.35	0.695	(0.376-1.286)	0.2466
>25.1	15	584	2675.97	560.54	0.823	(0.448-1.509)	0.5281
Cumulative duration of rosiglitazone therapy treated as a continuous variable							
For every 1-month increment of rosiglitazone use					0.993	(0.979-1.008)	0.3883

*n*: incident cases of dementia, *N*: cases followed, HR: hazard ratio, CI: confidence interval

### Hazard ratios estimated for patients enrolled during different periods of time

Table 4 shows the overall hazard ratios for ever versus never users of rosiglitazone estimated for patients who were enrolled during three different periods of time (i.e., 1999-2000, 2001-2003 and 2004-2006). A null association between rosiglitazone use and dementia risk was consistently observed.

**Table 4:** Overall hazard ratios for dementia comparing ever versus never users of rosiglitazone in type 2 diabetes patients enrolled in three different periods of time

Year	Ever users of rosiglitazone			Never users of rosiglitazone			Cox regression		
	*n*	*N*		*n*	*N*		HR	95% CI	*P* value
1999-2000	50	1919		60	1694		0.759	(0.518-1.112)	0.1570
2001-2003	54	2199		45	1943		1.029	(0.686-1.543)	0.8901
2004-2006	17	930		22	1411		1.100	(0.556-2.175)	0.7852

*n*: incident cases of dementia, *N*: cases followed, HR: hazard ratio, CI: confidence interval

## DISCUSSION

The findings suggested that rosiglitazone use in patients with type 2 diabetes mellitus had a neutral effect on the risk of dementia (Tables 2-4).

It is interesting that the risk of dementia was decreased in association with the use of metformin [12] and pioglitazone [13] in our previous studies but not associated with rosiglitazone in the present study. Metformin [10] and pioglitazone [11] both cross the blood-brain barrier and may therefore reduce insulin resistance and inflammation in the brain. The reduced risk associated with pioglitazone may also result from the regulation of PPARγ on genes associated with Alzheimer’s disease [8]. One of the possible explanations for a null effect associated with rosiglitazone is that rosiglitazone does not cross the blood-brain barrier [3] or only in a small proportion of 9-14% [3,28]. Different genetic backgrounds may also affect the responses to a drug treatment. For example, an earlier study suggested that patients with mild to moderate Alzheimer’s disease and without ApoE4 allele might response to rosiglitazone treatment while those with such an allele would not response to the treatment [3]. Since we did not have genetic polymorphisms of ApoE for additional analyses, whether this might have explained the lack of a protective effect of rosiglitazone on dementia in our patients with type 2 diabetes mellitus remains to be answered. Following PPARγ activation, pioglitazone and rosiglitazone may affect the transcriptions of different sets of genes and such a discrepancy might have also explained their different effects on dementia risk and other clinical events. For example, pioglitazone has a beneficial effect on lipid profile [29,30] and cardiovascular events [31,32], but rosiglitazone may adversely affect lipid profile [30] and cardiovascular risk [14]. Therefore, it is not known whether different sets of genes with opposite effects on dementia may be regulated by pioglitazone and rosiglitazone, respectively. The different effects on dementia exerted by rosiglitazone and pioglitazone are worthy of more in-depth investigation for their potential clinical implications. A recent study showed that the brain concentration of pioglitazone is affected by P-glycoprotein, a drug efflux transporter [33]. While (+)-pioglitazone is more resistant to this efflux transporter and accumulates in higher concentrations in the brain tissue, (-)-pioglitazone is less resistant to the efflux transporter and accumulates less in the brain. It is also worthy to further investigate whether the brain concentration of rosiglitazone may be affected by the stereospecific types of rosiglitazone compounds.

While patients with atrial fibrillation may have a higher risk of dementia, some recent studies suggested that the use of oral anticoagulants may provide a protective effect against dementia [34,35]. An estimated risk reduction of 21% (relative risk 0.79, 95% confidence interval: 0.67-0.93) was associated with the use of oral anticoagulants in a meta-analysis that includes 1 randomized controlled trial and 5 observational studies [34]. An observational study also suggested that the protective effect might be more remarkable for the use of non-vitamin K antagonist oral anticoagulants (adjusted hazard ratio 0.48, 95% confidence interval: 0.40-0.58) than for vitamin K antagonist (adjusted hazard ratio 0.62, 95% confidence interval: 0.60-0.64) [35]. Because non-vitamin K antagonist oral anticoagulants were not available during the study period up to 2011 in Taiwan, only warfarin, a vitamin K antagonist, could be included as a potential confounder in the present study (Table 1). In secondary analyses we found that atrial fibrillation was significantly associated with an increased risk of dementia (adjusted hazard ratio 2.270, 95% confidence interval: 1.332-3.869, *P* = 0.0026) but the use of warfarin had a neutral effect after adjustment for all covariates including atrial fibrillation (adjusted hazard ratio 0.949, 95% confidence interval: 0.488-1.846, *P* = 0.8777). Therefore, whether the use of oral anticoagulants may reduce the risk of dementia requires additional analyses. In additional subgroup analyses in patients with and without a diagnosis of atrial fibrillation and in patients who used and did not use warfarin, respectively, the risk of dementia remained neutral and insignificant for ever versus never users of rosiglitazone (data not shown). These secondary analyses still supported a neutral effect of rosiglitazone and did not change the conclusions of the study.

This study may have some clinical and research significance even though rosiglitazone is no longer widely used in clinical practice. First, although insulin resistance may increase the risk of dementia [1–3], this study strongly supports that not all drugs that improve insulin resistance and lower blood glucose may have a beneficial effect on the prevention of dementia in humans. Second, neuroprotective findings of rosiglitazone observed in *in vitro*, *in vivo* and animal studies should not be readily interpreted as potential protection against dementia in humans without consideration of its accessibility to the brain. This may have important implications in the future development of insulin sensitizers for the treatment of dementia. Third, taking into account the potentially higher risk of cardiovascular disease associated with rosiglitazone use [14] and the lack of a beneficial effect on dementia (Tables 2-4), the usefulness of rosiglitazone as an oral antidiabetic drug requires further justification.

The study has some merits that deserve mentioning. Because the NHI database covers >99% of Taiwan’s population, the findings can be generalized to the whole population. The potential bias resulted from self-reporting could be much reduced by using the medical records. Detection bias due to different socioeconomic status is less likely in our healthcare system because drug cost-sharing is low and can always be waived in patients with low-income, in veterans and when the patients receive prescription refills for chronic disease.

Study limitations include a lack of blood levels of glucose and insulin and a lack of indicators of insulin resistance (especially in the brain) and β-cell function for more in-depth analyses. Furthermore, the information of some confounders like nutritional status, dietary pattern, lifestyle, exercise, anthropometric factors, smoking, alcohol drinking, family history, and genetic parameters was not available.

In summary, unlike our previous study that showed a beneficial effect of pioglitazone on the risk of dementia in patients with type 2 diabetes mellitus, the present study finds a neutral effect of rosiglitazone. The discrepant effects between rosiglitazone and pioglitazone are worthy of more in-depth investigation.

## MATERIALS AND METHODS

### NHI reimbursement database

Taiwan’s NHI is a unique healthcare system implemented since March 1995. It covers >99.6% of the population. The Bureau of NHI has contracts with all in-hospitals and 93% of all medical settings throughout the nation. The database keeps all records of disease diagnoses, medication prescriptions and performed procedures and can be used for academic research after ethics review. The database was described in more details in our previous papers [36,37] and this retrospective cohort study used the longitudinal reimbursement database of the NHI for analyses with an approved number of 99274.

### Selection of a propensity score-matched study cohort

**Figure 1:**
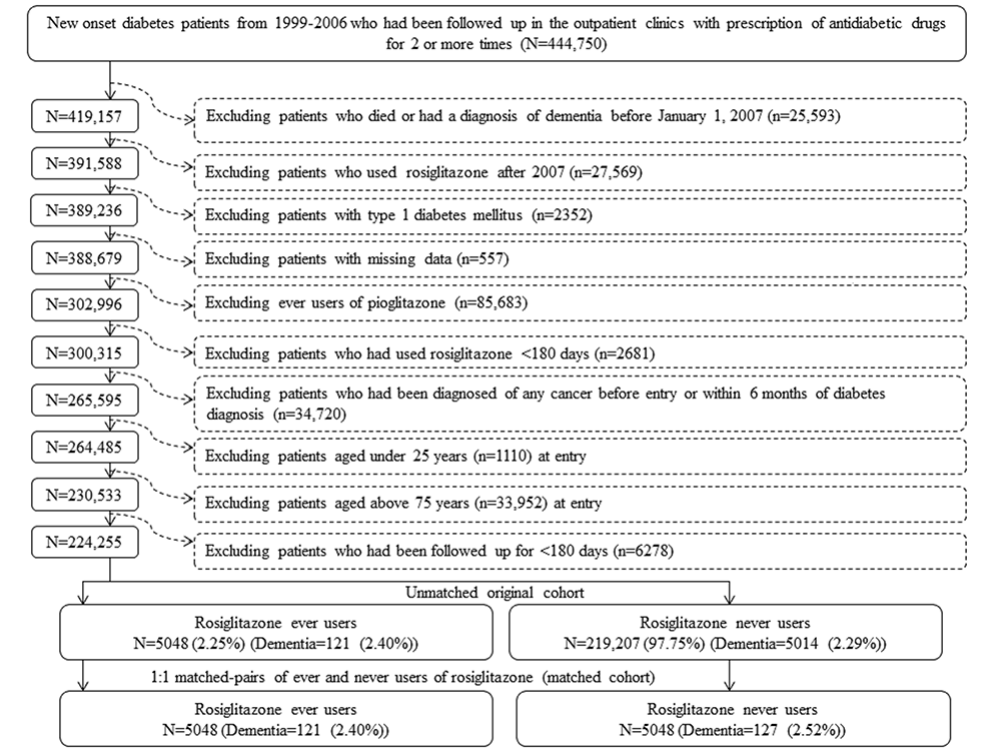
**Flowchart for the procedures in selecting a propensity score matched cohort of rosiglitazone ever users and never users.**

Figure 1 shows the procedures followed in the creation of a cohort of 1:1 matched-pairs of ever and never users of rosiglitazone used in the study. Because a meta-analysis published in 2007 suggested a potential risk of cardiovascular disease associated with rosiglitazone use [14], the prescription of rosiglitazone had been withdrawn in many patients in Taiwan not based on clinical judgment but because of psychological impacts. To avoid the potential impact of some unknown factors following this event, the present study restricted the enrollment of ever users of rosiglitazone to patients who used the drug before 2007. At first, 444,750 patients with new-onset diabetes mellitus during 1999-2006 and having been prescribed antidiabetic drugs for 2 or more times were identified from the outpatient clinics. Patients with a diagnosis of diabetes mellitus between 1996 and 1998 were not included to ensure a new diagnosis after 1999. The following ineligible patients were then excluded: 1) patients who died or had a diagnosis of dementia before January 1, 2007 (n=25,593), 2) patients who were initiated with rosiglitazone use after 2007 (n=27,569), 3) type 1 diabetes mellitus (n=2,352), 4) missing data (n=557); 5) ever users of pioglitazone (n=85,683), 6) rosiglitazone use for <180 days (n=2,681), 7) diagnosis of any cancer before entry or within 6 months of diabetes diagnosis (n=34,720, cancer patients might have a shortened lifespan and were excluded because they might have distorted follow-up time and dementia could be misdiagnosed from the clinical presentations of malignancy), 8) age <25 years (n=1,110), 9) age >75 years (n=33,952) and 10) follow-up duration <180 days (n=6,278). As a result, 5,048 ever users and 219,207 never users of rosiglitazone were identified as the unmatched original cohort. A cohort of 1:1 matched-pairs of 5,048 ever users and 5,048 never users (the matched cohort) was created by matching on propensity score (PS) based on the Greedy 8→1 digit match algorithm [38]. Logistic regression was used to create the PS with all characteristics listed in Table 1 being treated as independent variables.

### Definitions of variables

Throughout the study period, diabetes was coded 250.XX according to the International Classification of Diseases, Ninth Revision, Clinical Modification (ICD-9-CM) and dementia was coded as abridged codes of A210 or A222, or as ICD-9-CM codes of 290.0, 290.1, 290.2, 290.4, 294.1, 331.0–331.2, or 331.7–331.9.

Potential confounders were divided into the following categories: demographic data, major comorbidities associated with diabetes mellitus, diabetes-related complications, some major risk factors of dementia, potential risk factors of cancer, antidiabetic drugs, medications commonly used in diabetes patients and use of warfarin. Demographic data included age, sex, diabetes duration, occupation and living region (classified as Taipei, Northern, Central, Southern, and Kao-Ping/Eastern). Occupation was classified as class I (civil servants, teachers, employees of governmental or private businesses, professionals and technicians), class II (people without a specific employer, self-employed people or seamen), class III (farmers or fishermen) and class IV (low-income families supported by social welfare, or veterans). Atrial fibrillation was defined by ICD-9-CM code of 427.31 and the ICD-9-CM codes for other potential confounders for major comorbidities associated with diabetes mellitus (i.e., hypertension, dyslipidemia and obesity), diabetes-related complications (i.e., nephropathy, eye disease, stroke, ischemic heart disease and peripheral arterial disease), some major risk factors of dementia (i.e., head injury, Parkinson’s disease and hypoglycemia) and potential risk factors of cancer (chronic obstructive pulmonary disease, tobacco abuse and alcohol-related diagnoses) can be found in a previously published paper [13]. Antidiabetic drugs included insulin, sulfonylureas, metformin, meglitinide, and acarbose. Commonly used medications in diabetes patients included angiotensin converting enzyme inhibitors/angiotensin receptor blockers, calcium channel blockers, statins, fibrates, and aspirin. The only oral anticoagulant included was warfarin because all non-vitamin K antagonist oral anticoagulants were not available in Taiwan during the study period.

### Follow-up and calculation of incidence

Cumulative duration of rosiglitazone therapy was calculated in months from the database. Incidence density of dementia was calculated for the following subgroups of rosiglitazone use: never users, ever users and the tertiles of cumulative duration of therapy. The numerator was the case number of newly diagnosed dementia identified during follow-up and the denominator was the follow-up duration in person-years. Follow-up started on January 1, 2007 and ended on December 31, 2011, at the time of a new diagnosis of dementia, or on the date of death or the last reimbursement record, whichever occurred first.

### Main analyses

Standardized difference was calculated for each potential confounder as recommended by Austin and Stuart, who also suggested the use of a cutoff value of >10% as an indicator of potential confounding from the variable [39].

All potential confounders were adjusted for in Cox proportional hazards regression models, which were used to estimate the hazard ratios and their 95% confidence intervals for ever users and for each tertile of cumulative duration of rosiglitazone therapy in comparison to a referent group of never users. Cumulative duration of rosiglitazone therapy was also treated as a continuous variable for estimating hazard ratios for every 1-month increment of use. *P* < 0.05 was considered statistically significant for the hazard ratios.

### Subgroup analyses

To further examine whether the effect of rosiglitazone could be independent of metformin use, the above Cox proportional hazards regression models were also performed separately in subgroups of patients of ever and never users of metformin.

Before 1995, only metformin and sulfonylureas were available as oral antidiabetic drugs in Taiwan. In consideration that more antidiabetic drugs were available after 1995 and the guidelines for the use of antidiabetic drugs have evolved following the introduction of newer classes of drugs, the hazard ratios for dementia for ever versus never users of rosiglitazone were also estimated for patients whose diabetes was diagnosed during three different periods of time: 1999-2000, 2001-2003 and 2004-2006.

### Statistical software

Analyses were conducted using SAS statistical software, version 9.4 (SAS Institute, Cary, NC).

## References

[1] Steen E, Terry BM, Rivera EJ, Cannon JL, Neely TR, Tavares R, Xu XJ, Wands JR, de la Monte SM. Impaired insulin and insulin-like growth factor expression and signaling mechanisms in Alzheimer’s disease--is this type 3 diabetes? J Alzheimers Dis. 2005; 7:63–80. 10.3233/JAD-2005-7107 15750215

[2] de la Monte SM, Tong M, Wands JR. The 20-year voyage aboard the Journal of Alzheimer’s Disease: docking at ‘type 3 diabetes’, environmental/exposure factors, pathogenic mechanisms, and potential treatments. J Alzheimers Dis. 2018; 62:1381–90. 10.3233/JAD-170829 29562538 PMC5870020

[3] Risner ME, Saunders AM, Altman JF, Ormandy GC, Craft S, Foley IM, Zvartau-Hind ME, Hosford DA, Roses AD, and Rosiglitazone in Alzheimer’s Disease Study Group. Efficacy of rosiglitazone in a genetically defined population with mild-to-moderate Alzheimer’s disease. Pharmacogenomics J. 2006; 6:246–54. 10.1038/sj.tpj.6500369 16446752

[4] Li X, Song D, Leng SX. Link between type 2 diabetes and Alzheimer’s disease: from epidemiology to mechanism and treatment. Clin Interv Aging. 2015; 10:549–60. 10.2147/CIA.S74042 25792818 PMC4360697

[5] Willem M, Tahirovic S, Busche MA, Ovsepian SV, Chafai M, Kootar S, Hornburg D, Evans LD, Moore S, Daria A, Hampel H, Müller V, Giudici C, et al. η-Secretase processing of APP inhibits neuronal activity in the hippocampus. Nature. 2015; 526:443–47. 10.1038/nature14864 26322584 PMC6570618

[6] Villapol S. Roles of peroxisome proliferator-activated receptor gamma on brain and peripheral inflammation. Cell Mol Neurobiol. 2018; 38:121–32. 10.1007/s10571-017-0554-5 28975471 PMC5776063

[7] d’Abramo C, Massone S, Zingg JM, Pizzuti A, Marambaud P, Dalla Piccola B, Azzi A, Marinari UM, Pronzato MA, Ricciarelli R. Role of peroxisome proliferator-activated receptor gamma in amyloid precursor protein processing and amyloid beta-mediated cell death. Biochem J. 2005; 391:693–98. 10.1042/BJ20050560 15946122 PMC1276971

[8] Barrera J, Subramanian S, Chiba-Falek O. Probing the role of PPARγ in the regulation of late-onset Alzheimer’s disease-associated genes. PLoS One. 2018; 13:e0196943. 10.1371/journal.pone.0196943 29723294 PMC5933777

[9] Watson GS, Craft S. The role of insulin resistance in the pathogenesis of Alzheimer’s disease: implications for treatment. CNS Drugs. 2003; 17:27–45. 10.2165/00023210-200317010-00003 12467491

[10] Łabuzek K, Suchy D, Gabryel B, Bielecka A, Liber S, Okopień B. Quantification of metformin by the HPLC method in brain regions, cerebrospinal fluid and plasma of rats treated with lipopolysaccharide. Pharmacol Rep. 2010; 62:956–65. 10.1016/S1734-1140(10)70357-1 21098880

[11] Grommes C, Karlo JC, Caprariello A, Blankenship D, Dechant A, Landreth GE. The PPARγ agonist pioglitazone crosses the blood-brain barrier and reduces tumor growth in a human xenograft model. Cancer Chemother Pharmacol. 2013; 71:929–36. 10.1007/s00280-013-2084-2 23358645

[12] Chin-Hsiao T. Metformin and the risk of dementia in type 2 diabetes patients. Aging Dis. 2019; 10:37–48. 10.14336/AD.2017.1202 30705766 PMC6345339

[13] Tseng CH. Pioglitazone reduces dementia risk in patients with type 2 diabetes mellitus: a retrospective cohort analysis. J Clin Med. 2018; 7:E306. 10.3390/jcm7100306 30262775 PMC6209987

[14] Nissen SE, Wolski K. Effect of rosiglitazone on the risk of myocardial infarction and death from cardiovascular causes. N Engl J Med. 2007; 356:2457–71. 10.1056/NEJMoa072761 17517853

[15] Xu H, Chen X, Wang J, Yang T, Liu N, Cheng J, Gao R, Liu J, Xiao H. Involvement of insulin signalling pathway in methamphetamine-induced hyperphosphorylation of Tau. Toxicology. 2018; 408:88–94. 10.1016/j.tox.2018.07.002 29981415

[16] Li H, Wu J, Zhu L, Sha L, Yang S, Wei J, Ji L, Tang X, Mao K, Cao L, Wei N, Xie W, Yang Z. Insulin degrading enzyme contributes to the pathology in a mixed model of Type 2 diabetes and Alzheimer’s disease: possible mechanisms of IDE in T2D and AD. Biosci Rep. 2018; 38:BSR20170862. 10.1042/BSR20170862 29222348 PMC6435468

[17] Omeragic A, Hoque MT, Choi UY, Bendayan R. Peroxisome proliferator-activated receptor-gamma: potential molecular therapeutic target for HIV-1-associated brain inflammation. J Neuroinflammation. 2017; 14:183. 10.1186/s12974-017-0957-8 28886715 PMC5591559

[18] Hsu WJ, Wildburger NC, Haidacher SJ, Nenov MN, Folorunso O, Singh AK, Chesson BC, Franklin WF, Cortez I, Sadygov RG, Dineley KT, Rudra JS, Taglialatela G, et al. PPARgamma agonists rescue increased phosphorylation of FGF14 at S226 in the Tg2576 mouse model of Alzheimer’s disease. Exp Neurol. 2017; 295:1–17. 10.1016/j.expneurol.2017.05.005 28522250 PMC5762202

[19] Chiang MC, Cheng YC, Nicol CJ, Lin KH, Yen CH, Chen SJ, Huang RN. Rosiglitazone activation of PPARγ-dependent signaling is neuroprotective in mutant huntingtin expressing cells. Exp Cell Res. 2015; 338:183–93. 10.1016/j.yexcr.2015.09.005 26362846

[20] Jahrling JB, Hernandez CM, Denner L, Dineley KT. PPARγ recruitment to active ERK during memory consolidation is required for Alzheimer’s disease-related cognitive enhancement. J Neurosci. 2014; 34:4054–63. 10.1523/JNEUROSCI.4024-13.2014 24623782 PMC3951699

[21] Mishra J, Chaudhary T, Kumar A. Rosiglitazone synergizes the neuroprotective effects of valproic acid against quinolinic acid-induced neurotoxicity in rats: targeting PPARγ and HDAC pathways. Neurotox Res. 2014; 26:130–51. 10.1007/s12640-014-9458-z 24566814

[22] Escribano L, Simón AM, Gimeno E, Cuadrado-Tejedor M, López de Maturana R, García-Osta A, Ricobaraza A, Pérez-Mediavilla A, Del Río J, Frechilla D. Rosiglitazone rescues memory impairment in Alzheimer’s transgenic mice: mechanisms involving a reduced amyloid and tau pathology. Neuropsychopharmacology. 2010; 35:1593–604. 10.1038/npp.2010.32 20336061 PMC3055461

[23] Rodriguez-Rivera J, Denner L, Dineley KT. Rosiglitazone reversal of Tg2576 cognitive deficits is independent of peripheral gluco-regulatory status. Behav Brain Res. 2011; 216:255–61. 10.1016/j.bbr.2010.08.002 20709114 PMC2975872

[24] Watson GS, Cholerton BA, Reger MA, Baker LD, Plymate SR, Asthana S, Fishel MA, Kulstad JJ, Green PS, Cook DG, Kahn SE, Keeling ML, Craft S. Preserved cognition in patients with early Alzheimer disease and amnestic mild cognitive impairment during treatment with rosiglitazone: a preliminary study. Am J Geriatr Psychiatry. 2005; 13:950–58. 10.1097/00019442-200511000-00005 16286438

[25] Harrington C, Sawchak S, Chiang C, Davies J, Donovan C, Saunders AM, Irizarry M, Jeter B, Zvartau-Hind M, van Dyck CH, Gold M. Rosiglitazone does not improve cognition or global function when used as adjunctive therapy to AChE inhibitors in mild-to-moderate Alzheimer’s disease: two phase 3 studies. Curr Alzheimer Res. 2011; 8:592–606. 10.2174/156720511796391935 21592048

[26] Tzimopoulou S, Cunningham VJ, Nichols TE, Searle G, Bird NP, Mistry P, Dixon IJ, Hallett WA, Whitcher B, Brown AP, Zvartau-Hind M, Lotay N, Lai RY, et al. A multi-center randomized proof-of-concept clinical trial applying [^18^F]FDG-PET for evaluation of metabolic therapy with rosiglitazone XR in mild to moderate Alzheimer’s disease. J Alzheimers Dis. 2010; 22:1241–56. 10.3233/JAD-2010-100939 20930300

[27] Gold M, Alderton C, Zvartau-Hind M, Egginton S, Saunders AM, Irizarry M, Craft S, Landreth G, Linnamägi U, Sawchak S. Rosiglitazone monotherapy in mild-to-moderate Alzheimer’s disease: results from a randomized, double-blind, placebo-controlled phase III study. Dement Geriatr Cogn Disord. 2010; 30:131–46. 10.1159/000318845 20733306 PMC3214882

[28] Strum JC, Shehee R, Virley D, Richardson J, Mattie M, Selley P, Ghosh S, Nock C, Saunders A, Roses A. Rosiglitazone induces mitochondrial biogenesis in mouse brain. J Alzheimers Dis. 2007; 11:45–51. 10.3233/JAD-2007-11108 17361034

[29] Tseng CH, Huang TS. Pioglitazone with sulfonylurea: glycemic and lipid effects in Taiwanese type 2 diabetic patients. Diabetes Res Clin Pract. 2005; 70:193–94. 10.1016/j.diabres.2004.11.003 16188579

[30] Barnett AH. Redefining the role of thiazolidinediones in the management of type 2 diabetes. Vasc Health Risk Manag. 2009; 5:141–51. 10.2147/VHRM.S4664 19436665 PMC2672454

[31] Dormandy JA, Charbonnel B, Eckland DJ, Erdmann E, Massi-Benedetti M, Moules IK, Skene AM, Tan MH, Lefèbvre PJ, Murray GD, Standl E, Wilcox RG, Wilhelmsen L, et al, and PROactive Investigators. Secondary prevention of macrovascular events in patients with type 2 diabetes in the PROactive Study (PROspective pioglitAzone Clinical Trial In macroVascular Events): a randomised controlled trial. Lancet. 2005; 366:1279–89. 10.1016/S0140-6736(05)67528-9 16214598

[32] Kernan WN, Viscoli CM, Furie KL, Young LH, Inzucchi SE, Gorman M, Guarino PD, Lovejoy AM, Peduzzi PN, Conwit R, Brass LM, Schwartz GG, Adams HP Jr, et al, and IRIS Trial Investigators. Pioglitazone after ischemic stroke or transient ischemic attack. N Engl J Med. 2016; 374:1321–31. 10.1056/NEJMoa1506930 26886418 PMC4887756

[33] Chang KL, Pee HN, Yang S, Ho PC. Influence of drug transporters and stereoselectivity on the brain penetration of pioglitazone as a potential medicine against Alzheimer’s disease. Sci Rep. 2015; 5:9000. 10.1038/srep09000 25760794 PMC5390903

[34] Mongkhon P, Naser AY, Fanning L, Tse G, Lau WC, Wong IC, Kongkaew C. Oral anticoagulants and risk of dementia: A systematic review and meta-analysis of observational studies and randomized controlled trials. Neurosci Biobehav Rev. 2019; 96:1–9. 10.1016/j.neubiorev.2018.10.025 30391408

[35] Friberg L, Rosenqvist M. Less dementia with oral anticoagulation in atrial fibrillation. Eur Heart J. 2018; 39:453–60. 10.1093/eurheartj/ehx579 29077849

[36] Tseng CH. Metformin and lung cancer risk in patients with type 2 diabetes mellitus. Oncotarget. 2017; 8:41132–42. 10.18632/oncotarget.17066 28456789 PMC5522244

[37] Tseng CH. Metformin is associated with a lower risk of colorectal cancer in Taiwanese patients with type 2 diabetes: A retrospective cohort analysis. Diabetes Metab. 2017; 43:438–45. 10.1016/j.diabet.2017.03.004 28438547

[38] Parsons LS. Performing a 1:N case-control match on propensity score. http://www2.sas.com/proceedings/sugi29/165-29.pdf (last accessed March 23, 2019).

[39] Austin PC, Stuart EA. Moving towards best practice when using inverse probability of treatment weighting (IPTW) using the propensity score to estimate causal treatment effects in observational studies. Stat Med. 2015; 34:3661–79. 10.1002/sim.6607 26238958 PMC4626409

